# A New Argument for the Groundedness of Grounding Facts

**DOI:** 10.1007/s10670-021-00416-7

**Published:** 2021-06-08

**Authors:** Fabrice Correia

**Affiliations:** Department of Philosophy, University of Geneva, Rue De-Candolle 5, 1211 Geneva 4, Switzerland

## Abstract

Many philosophers have recently been impressed by an argument to the effect that all grounding facts about “derivative entities”—e.g. the facts expressed by the (let us suppose) true sentences ‘the fact that Beijing is a concrete entity is grounded in the fact that its parts are concrete’ and ‘the fact that there are cities is grounded in the fact that p’, where ‘p’ is a suitable sentence couched in the language of particle physics—must themselves be grounded. This argument relies on a principle, *Purity*, which states that facts about derivative entities are non-fundamental. Purity is questionable. In this paper, I introduce a new argument—the argument from *Settledness*—for a similar conclusion but which does not rely on Purity. The conclusion of the new argument is that every “thick” grounding fact is grounded, where a grounding fact [F is grounded in G, H, …] is said to be thick when at least one of F, G, H, … is a fact—a condition that is automatically satisfied if grounding is factive. After introducing the argument, I compare it with the argument from Purity, and I assess its cogency relative to the relevant accounts of the connections between grounding and fundamentality that are available in the literature.

## The argument from Purity

Following currently accepted terminology, let me call ‘grounding facts’ the facts that can be picked out by means of expressions of type ‘the fact that (⋅⋅⋅ is grounded in –-)’.^1^ There is a now familiar way of arguing for the view that grounding facts about “derivative entities”—entities such as cities, people, molecules and planets, say—must themselves be grounded. This way of arguing, I take it, is faithfully captured by the following particular argument—*the argument from Purity*, as I will call it:^2^

1) Every fact about derivative entities is non-fundamental: Purity
2) Every grounding fact about derivative entities is non-fundamental: By 1)
3) If a fact is non-fundamental, then it is grounded: NF→G
4) Every grounding fact about derivative entities is grounded: By 2) and 3)

Given that there are facts about derivative entities (e.g. the fact that there are cities, or the fact that Beijing is bigger than Paris), the premises of the argument guarantee that there are grounding facts about derivative entities, and hence that the conclusion is *non-vacuously* true.^3^

Purity owes its name to a principle Ted Sider (2011) calls by the same name, which states that no truth involving non-fundamental notions is fundamental. On the assumption that all facts about derivative entities are truths involving non-fundamental notions, Sider’s principle implies mine. Although it is natural to take this assumption for granted given the way Sider understands and uses his principle, one can stay neutral on the issue: the principle that is relevant for the argument from Purity as it is commonly accepted is the one that appears as premise 1) above—or so I take it.

The argument from Purity poses a serious challenge. For what are the grounding facts about (say) cities grounded in? In some cases, the answer is easy—or so it may seem. Thus, given the orthodox view that grounding is transitive, it is perhaps plausible to hold that.

- The fact that [{{Beijing}} exists] is grounded in [Beijing exists]

is itself grounded in

- The fact that [{{Beijing}} exists] is grounded in [{Beijing} exists]

and

- The fact that [{Beijing} exists] is grounded in [Beijing exists].^4^

Yet many other cases are different. Assume for example the physicalist view that some given microphysical fact grounds the fact that there are cities. What grounds this grounding fact? There is no obvious answer.

Many philosophers have been impressed by this argument, and several, more or less plausible suggestions about what grounds grounding facts have been put forward in order to accommodate its conclusion.^5^ It is not my aim in this paper to assess these suggestions. Nor is it my intention to attack the argument from Purity. What I want to do is put forward a new argument of the same kind, compare it with the argument from Purity, and assess its cogency relative to the relevant conceptions of the connections between grounding and fundamentality that can be found in the literature. Before doing that, it will prove useful to elaborate a bit on the argument from Purity.

## Some Remarks About the Argument from Purity

The argument from Purity involves three key notions: a fact’s being about derivative entities, being fundamental, and grounding. Each of these notions is equivocal in the present context, and therefore, before critically examining the argument, one should in principle specify how these notions are to be understood. It can be argued that specifying how the notion of a fact’s being about derivative entities is to be understood is not really mandatory. Instead of critically examining the argument from Purity itself, one may as well critically examine the arguments that result from it by replacing all occurrences of ‘derivative entities’ by kind terms that pick out entities that presumably count as derivative, e.g. ‘cities’, ‘people’, ‘molecules’ or ‘planets’. By moving from the “general” argument from Purity to particular “instances” in the suggested way, something is lost, for sure, but for practical purposes this may not matter.^6^ By contrast, something definitely needs to be said about the other two notions.

It is common to distinguish between various notions of grounding, e.g. between factive and non-factive, weak and strict, and worldly and representational grounding.^7^ A full assessment of the argument from Purity should in principle specify which notion, or range of notions, is supposed to be under focus. Some notions must clearly be discarded as being unintended, unconditionally or in combination with certain assumptions.

Suppose for instance that the argument is understood as involving *weak* grounding. Since weak grounding is reflexive, the conclusion of the argument from Purity is trivial, and therefore the argument as a whole is obviously dialectically ineffective. Proponents of the argument are thus best understood as having *strict* grounding in mind.

Also, suppose that the argument is understood as involving a *factive* notion grounding, i.e. that grounding is understood in such a way that it is impossible that grounding be exemplified without the exemplifiers holding, i.e. without the exemplifiers being facts.^8^ Suppose in addition that this notion is analysable in terms of a non-factive notion along the following lines: item F is factively grounded in item(s) FF just in case (1) FF hold/s and (2) F is non-factively grounded in FF. Then the conclusion of the argument from Purity is highly plausible, if not trivial, because it is highly plausible, if not trivial, that [FF hold/s & F is non-factively grounded in FF], if it obtains, is grounded (both non-factively and factively) in [FF hold/s] and [F is non-factively grounded in FF] taken together. Proponents of the argument are thus best understood as having in mind either a non-factive notion of grounding, or a factive notion that is not analysable in terms of a non-factive notion in the suggested way.

These considerations leave open the possibility of there being more than one notion of grounding that the argument from Purity can be taken to target. It is also plausible to hold that ‘fundamental’ may be understood in different ways in the argument. Knowledge of the literature on fundamentality naturally suggests the following four candidate notions: being “natural” or “joint-carving” (à la Lewis, 1983 and Sider, 2011), being ungrounded (Bennett, ; Rosen, 2010; Schaffer, 2009 and many more), being “all-grounding” (see Leuenberger, 2020 for an illuminating discussion) and not being less fundamental than other facts (an option which, surprisingly, is hardly ever explicitly mentioned—but see Tahko, 2018). A proper assessment of the argument from Purity should in principle specify which notion of fundamentality, or range of such notions, is supposed to be involved. None of these notions, it seems, should be *clearly* discarded as being unintended.

That the choice of a notion among the various admissible candidates is important is particularly evident in the case of fundamentality. Suppose for instance that the notion of fundamentality at work in the argument is that of ungroundedness. Then NF → G is guaranteed to be true, but Purity is questionable (here and below, I use ‘questionable’ in the sense of ‘open to dispute’, not as implying that what is questionable must be dismissed). For consider [NYC = NYC]. This is a fact about a city, and hence (presumably) it counts as a fact about derivative entities. By Purity, it is non-fundamental, i.e. (given the notion of fundamentality at stake) grounded. But is it? [NYC = NYC] look very much like a good candidate for being brute, i.e. ungrounded. I do not deny that there are accounts on which this fact is grounded. For instance, friends of grounding who espouse Sider’s conception of fundamentality might suggest that [NYC = NYC] is grounded in [def(NYC) = def(NYC)], where ‘def(NYC)’ is a definite description that provides a “metaphysical definition” of NYC couched in joint-carving terms (see Sider, 2011, Ch. 7). Or again, consider the view, put forward in Rosen (2010, p. 119), that if it is essential to something that p, then this fact grounds the fact that p. Assuming that every object is essentially self-identical, the view implies that [NYC = NYC] is grounded in [It is essential to NYC that NYC = NYC].^9^^,^ 10 But all the accounts I can think of are open to dispute,^11^ and hence support rather than undermine my claim that Purity, given the notion of fundamentality at stake, is itself questionable.^12^

Alternatively, suppose that the notion of fundamentality at work in the argument from Purity is the Siderian notion of being joint-carving. Then Purity has a great plausibility, and indeed Sider (2011) makes a good case for it. But if on the present understanding of the argument Purity is taken for granted, then NF → G is now as questionable as Purity was on the previous understanding of ‘fundamental’. For consider again [NYC = NYC]. This is a fact about a city, and hence a fact about derivative entities, and so by Purity it is non-fundamental. By NF → G, it follows that [NYC = NYC] is grounded. But as we just saw, the view that [NYC = NYC] is grounded is questionable. Thus, assuming that given the notion of fundamentality at work in the argument, Purity is secured, we reach the conclusion that NF → G is questionable.

These last remarks suggest a general evaluative comment on the argument from Purity. For the argument from Purity to be sound, both Purity and NF → G must hold. Their conjunction yields the following Purity-like principle:

(Purity*) Every fact about derivative entities is grounded.

Using again [NYC = NYC] as I did before, one can argue that Purity* is questionable: granted that [NYC = NYC] is a fact about derivative entities, Purity* entails that it is grounded, but the view that it is grounded is questionable. The point is interesting, because it is independent from the particular notion of fundamentality taken to be involved in Purity and NF → G.

## The argument from Settledness

Let a *thick* grounding fact be a grounding fact [F is grounded in FF] such that F or one of FF holds, i.e. is a fact. If grounding is factive, then all grounding facts are thick, but if grounding is not factive, then there may be—indeed, there certainly are—grounding facts that are not thick. Consider, then, the following argument—the *argument from Settledness*, as I will call it:

1) For every thick grounding fact [F is grounded in FF] and some fact X among F and FF, [F is grounded in FF] is less fundamental than X: Settledness
2) A fact is non-fundamental if it is less fundamental than another fact: Truism
3) Every thick grounding fact is non-fundamental: By 1) and 2)
4) If a fact is non-fundamental, then it is grounded: NF→G
5) Every thick grounding fact is grounded: By 3) and 4)

Note that by Truism, NF → G and the assumption that some fact is less fundamental than some other fact, there are thick grounding facts and hence 5) is not vacuously true. Also note that if grounding is factive, then Settledness is equivalent to

- For every grounding fact [F is grounded in FF] and some X among F and FF, [F is grounded in FF] is less fundamental than X,

and the conclusion of the argument is equivalent to

- Every grounding fact is grounded.

The argument from Settledness is in some ways similar to the argument from Purity, and in some ways different. If grounding is taken to be factive, the conclusion of the argument from Settledness (that every grounding fact is grounded) is logically stronger than the conclusion of the argument from Purity (that every grounding fact about derivative entities is grounded). This makes the former argument at least as challenging as the latter.^13^ If grounding is *not* taken to be factive, the conclusion of the argument from Settledness (that every *thick* grounding fact is grounded) is not logically stronger than the conclusion of the argument from Purity, but it remains at least as challenging as the latter when the focus is on thick grounding facts.

The premises of the two arguments overlap. Like the argument from Purity, the new argument invokes NF → G. But unlike the former, it does not invoke Purity—nor any principle involving the notion of a fact’s being about derivative entities, for that matter. It has Settledness and Truism instead. Settledness, like Purity, connects grounding and fundamentality. But whereas Purity connects grounding and *absolute* fundamentality (the notion of being fundamental *tout court*), Settledness connects grounding and *relative* fundamentality (the notion of being less fundamental than). Truism connects absolute and relative fundamentality. Relative fundamentality is completely absent from the argument from Purity (at least, it does not appear there *explicitly*).

Importantly, the premises of the argument from Settledness do not entail Purity*. As a result, the questionable character of Purity* (see Sect. 2) is of no concern here: one may consistently find the new argument compelling and doubt or even reject Purity*. More generally, one may consistently be convinced by new argument and doubt or even reject the conjunction of the premises of the argument from Purity. This I take to be one good thing, dialectically speaking, about having the argument from Settledness in addition to the argument from Purity in our dialectical toolkit. These considerations are of course compatible with the view—which strikes me as correct—that the argument from Settledness may also consistently be taken to be sound by people who already accept the argument from Purity. For anyone already convinced by the argument from Purity who becomes convinced by the argument from Settledness, the latter simply adds grist to the mill.

Is the argument from Settledness compelling? The new premises, Truism and Settledness, are certainly *prima facie* convincing—or so it seems to me. Truism sounds really like, well, a truism: if a fact F is less fundamental than some other facts, then how could F possibly be fundamental? To appreciate this, it is important to properly understand the principle. Just like ‘fundamental’ may be understood in various ways, as expressing various properties of facts, ‘less fundamental than’ may also be understood as expression various relations between facts. It is easy to select an interpretation of ‘non-fundamental’ and an interpretation ‘less fundamental than’ in such a way that Truism, understood accordingly, is false, at least given certain background assumptions.^14^ The proper interpretation of Truism involves a notion of relative fundamentality and a notion of absolute fundamentality that are appropriately paired. I assume indeed, as I think it is reasonable to do, that for each notion of relative fundamentality, there is a sister notion of absolute fundamentality that naturally goes with it. I simply take Truism to record a core relation between the notion of relative fundamentality it involves and the corresponding sister notion of absolute fundamentality.^15^

Settledness may not sound literally truistic, but it seems nevertheless very plausible. The stronger view that

(Strong Settledness): For every thick grounding fact [F is grounded in FF] and *every* (not just some) fact X among F and FF, [F is grounded in FF] is less fundamental than X

sounds indeed compelling. However, these are only first impressions, and a proper assessment of the cogency of the argument requires at least prior specification of which key notions are involved.

The discussion of Sect. 2 is relevant here. There I pointed to the fact that there are various notions of grounding and various notions of absolute fundamentality, and I illustrated the point. Since the argument from Settledness invokes both grounding and absolute fundamentality, this variety should be taken into consideration in the present context, too. I also emphasised that the argument from Purity is best understood as concerning a notion of grounding that is strict and that is either non-factive, or factive but not analysable in terms of a non-factive notion in the way suggested there. This is equally true of the argument from Settledness. Finally, the discussion of NF → G is obviously pertinent. Depending on which notion of fundamentality is taken to be involved, the status of NF → G may change. To illustrate, if the notion of fundamentality involved in the argument is that of ungroundedness, then NF → G is guaranteed to be true, whereas if the notion involved is that of being joint-carving, then NF → G may be argued to be questionable. Given the view that [NYC = NYC] is non-fundamental—which is a consequence of Purity, but which may be independently supported—and the view—defended in Sect. 2—that the claim that [NYC = NYC] is grounded is questionable, NF → G is indeed questionable.

However, since the argument from Settledness involves the relative notion of being less fundamental than, the discussion of Sect. 2 is of significantly limited scope in the present context. Given that there are different notions of absolute fundamentality, it is to be expected that there are different notions of relative fundamentality. How relative fundamentality should be understood in the argument is clearly crucial, since it appears in the two new premises Settledness and Truism. What I would like to do in the remainder of this paper is to determine whether the argument from Settledness is cogent given the accounts of the connections between grounding, being fundamental and being less fundamental than that can be found in the literature.

## The Argument from Settledness and Existing Accounts of the Connections Between Grounding, Being Fundamental and Being more Fundamental than

This will actually not take too much time. For whereas the connections between grounding and *absolute* fundamentality have been reasonably thoroughly studied in the past few years, philosophical theorising on the connections between grounding, absolute fundamentality and *relative* fundamentality is greatly underdeveloped. As far as I know, there currently are only three reasonably elaborated views about these connections, one put forward in Bennett, (2017), one put forward in Werner (2020), and the third one explored in Correia (2021).^16^ Let me examine each in turn.

Bennett relativises fundamentality to “building relations”, and she takes grounding to be among these relations. She holds that one can characterise, in terms of each such relation, both a notion of being fundamental and a notion of being more fundamental than. For grounding, the characterisations go as follows (see Ch. 5 and Ch.6):^17^

- A fact is fundamental ≡_df_ it is ungrounded.
- Fact F is more fundamental than fact G ≡_df_ at least one of the following conditions is satisfied:
  (1): F is fewer grounding steps away from the ungrounded fact(s) that terminate its unique grounding chain than G is from the ungrounded fact(s) that terminate its unique grounding chain;
  (2): F at least partially grounds G;
  (3): F stands in the ancestral of partial grounding to G;
  (4): F is ungrounded while G is grounded;
  (5): F belongs to some kind K and G belongs to some kind K* such that
    - Neither K nor K* includes both grounded and ungrounded members, and
    - G does not belong to K and F does not belong to K*, and
    - K*s are typically or normally grounded in Ks.

Some parts of the second characterisation would require further elaboration in order to be fully clear, but we do not need to go into such details.^18^

Bennett’s characterisation of absolute fundamentality trivially validates NF → G. Moreover, a quick inspection of the five clauses of Bennett’s characterisation of being more fundamental than reveals that each clause entails ‘G is grounded’. Accordingly, an immediate consequence of the characterisation is that

(LF→G): If a fact is less fundamental than another fact, then it is grounded.

Putting the two characterisations together, we accordingly get Truism.^19^ Thus, Bennett’s account supports two of the three premises of the argument. However, given LF → G, 5) (namely, the conclusion of the argument from Settledness) directly follows from Settledness, and as a consequence, Bennett’s characterisation of the relation of being more fundamental than makes the argument dialectically ineffective: there cannot be a situation in which a minimally rational thinker who accepts Bennett’s characterisation would initially be willing to accept Settledness but not 5), and later on be moved by the argument to accepting 5).

The same diagnosis holds if Werner’s (2020) account of being more fundamental than is taken for granted instead. The account is technically involved, but the core idea is simple to state: it consists in taking a formally precise version of condition (1) in Bennett’s account as being not only sufficient but also necessary for a fact F to be more fundamental than a fact G. As a result, LF → G immediately follows from Werner’s account, just like it does on Bennett’s account, and accordingly, as announced, the account makes the argument from Settledness dialectically ineffective.

The situation with Correia (2021) is significantly different. In that paper, I explore an account of grounding in terms of a primitive relation of being more fundamental than. On the proposed account, F is grounded in FF just in case (i) FF hold/s, and (ii) the holding of FF entails that F holds and is less fundamental than FF. The notion of grounding so characterised is thus factive, and analysable in terms of a non-factive notion that is characterised by condition (ii) alone. If grounding is understood in the way suggested by this account, then the conclusion of the argument from Settledness sounds trivial. As a consequence, it is best to take the notion of grounding in the argument to be the non-factive notion characterised by (ii) (see my remarks above about how grounding should be understood both in the argument from Purity and in the argument from Settledness). I will accordingly throughout the rest of this section assume that the relevant characterisation of grounding goes as follows:

(Grounding from Fundamentality): F is grounded in FF ≡_df_ the holding of FF entails that F holds and is less fundamental than FF.

In the original characterisation of factive grounding, ‘entails’ is left underspecified on purpose, in order to allow several precisifications among which one may make a choice. Various notions of entailment may indeed be deemed relevant for the purpose of characterising factive grounding. Of course, the same is true of non-factive grounding. Importantly, Grounding from Fundamentality does not entail LF → G, and accordingly the previous considerations about Bennett’s account and Werner’s account do not apply to the account under focus.^20^

In Correia (2021), no account of absolute fundamentality is put forward, but it is very natural in the proposed setting to stipulatively define ‘is fundamental’ as ‘is not less fundamental than other facts’, and I will therefore assume that this definition is part of the package. Given this definition, Truism is analytic. By contrast, the overall account per se does not force the decision for or against the other two premises. Let me discuss them in turn.

Interestingly, the status of NF → G can be seen to vary with substantial metaphysical assumptions that are independent from the account. Thus, suppose that there are fundamental facts, and that all the fundamental facts are physical facts. Consider then the physicalist view that every non-fundamental fact is grounded in fundamental facts. This combination of views validates NF → G, and it is, on the face of it, compatible with Grounding from Fundamentality.^21^ Alternatively, suppose again that there are fundamental facts, and that all the fundamental facts are physical facts, but this time consider the anti-physicalist view that mental facts are not physical facts and that some mental facts are ungrounded. This could be a view on which the mental facts are “emergent from” but not “realised by” the physical facts, where it is understood that while realisation—like grounding according to orthodoxy—entails *metaphysical* necessitation, emergence only entails *nomological* necessitation (see e.g. Baysan, 2019 on the distinction). In any case, the combination of views in question invalidates NF → G, and it is, on the face of it, compatible with Grounding from Fundamentality.

The discussion of Settledness is less straightforward. My aim here is to assess Settledness in the light of the account explored in Correia (2021), i.e. to determine whether this account supports Settledness, rather than to assess Settledness *tout court*. However, it will be useful to present and then briefly discuss an argument in its favour. As we will see, the premises of a modified version of this argument are supported by the account.

I previously claimed that the principle seems plausible. But what more could be said in its favour? Here is an argument that strikes me as having *prima facie* a certain force.^22^ It is a natural thought that (A) if F is grounded in FF, then F and FF are components of [F is grounded in FF]. Couple this view with the further natural view that (B) facts that are components of other facts are more fundamental than them, and you get Settledness. (You actually get *Strong* Settledness.) (A) is nothing but a consequence of the familiar view that facts expressed by atomic sentences are complex entities, composed of properties and relations and their exemplifiers. One may wish to argue in favour of (B) on the grounds that (u) if a fact is a component of another fact, then the former is less complex than the latter, and (v) less complex facts are more fundamental than more complex facts. However, one may accept (B) without accepting (v), e.g. because one do not believe that facts have degrees of complexity, or more generally because one do not believe that there is such a thing as a relation of being more complex than among facts.

This argument in favour of Settledness can be attacked in various ways.^23^ Against (A), some may claim that facts expressed by atomic sentences have no components at all (as in the view explored in Skyrms, 1981), or that grounding facts have only non-facts as constituents (along these lines, one could claim e.g. that the only components of the fact that [Beijing is a concrete entity] is grounded in [Beijing’s parts are concrete] are Beijing, its parts, the property of being concrete and the grounding relation). Against (B), some may rely on the view that certain complex entities are more fundamental than at least some of their components (as in Schaffer, 2010), and specifically hold that cases of this sort happen to undermine (B). A particularly convincing illustration runs as follows. Suppose that F is fundamental, and that G is “highly non-fundamental” (G might be the fact that the Dow Jones fell by more than 2000 points on 9 March 2020). Consider the fact F ∨ (G & ¬G), where it is assumed not only that there are the relevant operations on facts, but also that the application of an operation on given facts yields a fact with the inputs as components, so that in particular G is a component of F ∨ (G & ¬G). Since F is both fundamental and an immediate ground for F ∨ (G & ¬G), the latter is plausibly quite close to being fundamental, and is consequently more fundamental than G. Since G is a component of F ∨ (G & ¬G), (B) is false.

This counterexample to (B) suggests a modified argument in favour of Settledness, based on principles that are just like (A) and (B) but which invoke the concept of being an *immediate* component instead of that of being a component:

(A*): If F is grounded in FF, then F and FF are immediate components of [F is grounded in FF];
(B*): Facts that are immediate components of other facts are more fundamental than them.

(B*) is not subject to the counterexample to (B) discussed above, because G is not an *immediate* component of F ∨ (G & ¬G). Since (A*) and (B*) together entail Settledness (in fact, Strong Settledness), the modified principles can still be used to support Settledness, as announced.

Of course, this amended argument in favour of Settledness does not escape all the objections to the original argument mentioned above, and accordingly a proper defence of the argument would require more work. But as far as my current purposes are concerned it is not necessary to push the discussion further. What is important is that the argument has been identified, because it can indeed be used to show that the views about relative fundamentality that I put forward in Correia (2021) support Settledness.

The way I treat relative fundamentality there is indeed guided by the following principle, where the sentences in question are taken from certain regimented languages: if sentence S is an immediate part of sentence T, and if both sentences are true, then the fact expressed by S is more fundamental than the fact expressed by T.^24^ As a result, for instance, if ˹S & T˺ is true, then the fact it expresses is less fundamental than the fact expressed by S and the fact expressed by T, and if ˹S ∨ T˺ is true, then the fact it expresses is less fundamental than the fact expressed by S if S is true, and is less fundamental than the fact expressed by T if T is true. This guiding principle is not exactly (B*). But I took for granted that the structure of facts is mirrored by the structure of the sentences that express them in the relevant regimented languages, and given this assumption, (B*) follows from the principle. On the same assumption, (A*) is also secured.

Let me wrap up the discussion of Correia (2021). Purity follows from the natural account of absolute fundamentality—to wit, the account according to which to be fundamental is to be not less fundamental than other facts—on the approach defended there. Given the proposed account of the connection between grounding and relative fundamentality, NF → G is validated on some substantial metaphysical views (e.g. the physicalist view described above), but is ruled out on some other substantial metaphysical views (e.g. the anti-physicalist view described above). Finally, Settledness is a consequence of some principles that follow from the particular way in which I conceive of the relation of being more fundamental than in the paper.

The upshot is that, on some substantial metaphysical views at least, the argument from Settledness has a grip on the account I explore there. If this account and some such view are taken for granted, which stories about what grounds the thick grounding facts can be put forward? It is not the place here for a thorough discussion of the available options, but let me just briefly show that the view that grounding is superinternal, i.e. that every grounding fact [F is grounded in FF] is grounded in FF (see footnote 22), follows from Grounding from Fundamentality and from my approach to relative fundamentality, given certain assumptions about the logic of entailment. Consider the following five general principles, the first two about grounding and entailment and the last three only about entailment (symbolised as ⇒):

(Order): (FF hold/s & F is grounded in FF) ⇒ FF are/is more fundamental than [F is grounded in FF]
(Rigidity): If F is grounded in FF, then FF hold/s ⇒ F is grounded in FF
(Reflexivity): φ ⇒ φ
(Transitivity): φ ⇒ ψ, ψ ⇒ χ / φ ⇒ χ
(&-Intro): φ ⇒ ψ, φ ⇒ χ / φ ⇒ ψ & χ

The following argument establishes that these five principles, together with Grounding from Fundamentality, yield the superinternality of grounding:

Suppose that F is grounded in FF. By Rigidity, it follows that FF hold/s ⇒ F is grounded in FF. By Reflexivity and &-Intro, we then get that FF hold/s ⇒ (FF hold/s & F is grounded in FF). Thanks to Order and Transitivity, we can infer that FF hold/s ⇒ FF are/is more fundamental than [F is grounded in FF]. By Grounding from Fundamentality, this means that [F is grounded in FF] is grounded in FF.

The three logical principles are very weak. Order is very close to Settledness—once Settledness is accepted, it is indeed hard to see how Order could be rejected (modulo acceptance of the relevant notion of entailment). As I emphasised, my views on relative fundamentality support Settledness. Rigidity is not obvious, and it is natural to think that it is the weakest link in the argument. However, note that given Grounding from Fundamentality, the principle follows from the following further principle about entailment:

(Expansion): φ ⇒ ψ / φ ⇒ (φ ⇒ ψ)

Expansion is not as weak as Reflexivity, Transitivity and &-Intro. It is not validated in the celebrated system R of relevant logic, for instance. Yet it is validated in systems that are much weaker than classical logic, e.g. in another celebrated system of relevant logic, RM, which can be axiomatised as R plus the Mingle axiom φ ⇒ (φ ⇒ φ) (it turns out that RM can alternatively be axiomatised by adding Expansion rather than Mingle—see Dunn & Restall, 2002). The question of whether Expansion is acceptable in the context under discussion deserves further scrutiny.

Let me wrap up the discussion of this section. Assuming Bennett’s account of the connections between grounding and fundamentality—more precisely, assuming her characterisation of being more fundamental than in terms of grounding, the argument from Settledness is dialectically ineffective because given that characterisation, the conclusion of the argument directly follows from Settledness. The same is true if Werner’s account of being more fundamental than is assumed instead. By contrast, assuming the account of grounding in terms of being more fundamental than explored in Correia (2021), the natural stipulative definition of absolute fundamentality in terms of the latter relation and the approach to relative fundamentality I develop in that paper, the force of the argument depends on what extra, substantial metaphysical views are taken for granted. While on some such assumptions, the argument is unsound, on some other such assumptions each premise of the argument either is guaranteed to be true or follows from my approach to relative fundamentality.

## Conclusion

Theorising about the connections between grounding, absolute fundamentality and relative fundamentality is in its infancy. So far, Bennett (2017), Werner (2020) and Correia (2021) are the only reasonably developed accounts of these connections, but it makes little doubt further accounts will be put forward in the near future. It will be interesting to figure out, for each of these future accounts, whether the argument from Settledness has some force once the account is taken on board.

## Footnotes

1 I assume a predicate regimentation of grounding claims rather than an operator regimentation (see e.g. Correia 2010 on this distinction), but this is just for convenience and I take it that the paper could be rewritten without loss (or gain) assuming the other regimentation. I take the relata of grounding to be proposition-like entities, say propositions or states of affairs, but I do not decide in favour of a specific ontological category. I use ‘fact’ to qualify those among these proposition-like entities that *hold*, i.e. are true or obtain. I also follow grounding orthodoxy and assume that grounding is many-one rather than many-many (see e.g. Litland 2016 on this distinction). Going for the non-orthodox view would certainly affect the discussion, though I am not sure up to which point.

2 See Bennett 2011, Dasgupta 2014, deRosset 2013, Litland 2017 and Sider 2020.

3 It is tempting to expand the argument from Purity to reach the stronger conclusion that *every* grounding fact is grounded, as follows:

5) Grounded facts are derivative entities: H
6) Every grounding fact is about a derivative entity: By 5)
7) Every grounding fact is grounded: By 4) and 6)

However, premise H is far from being trivial and hence requires argument. For the sake of simplicity, I will focus on the argument from Purity in its non-expanded form.

4 Following standard notation, I use ‘[…]’ for ‘the fact that …’. I here mix the two expression types in order to improve readability.

5 See the references in footnote 2. Some of these suggestions will be relevant for Sect. 4 below. Karen Bennett (2011, p. 27) puts forward an argument from modal recombination for the view that all grounding facts are grounded (which she attributes to Johnathan Schaffer), but this argument has had less impact on the philosophical community. Anyway, it is very different from the argument from Purity, and I will leave it completely aside here. For the same reason, I will also leave aside arguments based on the idea that grounding facts are grounded because (1) grounding is analysable (as e.g. in the views explored in Correia 2013 and Correia & Skiles 2019) and (2) an *analysans* grounds its *analysandum*.

6 See Sider (2020), pp. 748–749 (fn 6), for a similar point.

7 On factive vs non-factive and weak vs strict, see Fine (2012). On worldly vs representational, see Correia (2010, 2020) and Fine (2017).

8 See footnote 1 on my understanding of ‘facts’.

9 Note that the essentialist fact that is mentioned as a ground is itself about a city. Is this fact grounded? If not, as e.g. Shamik Dasgupta (2014) would have it, then Purity is still threatened. An alternative to the Rosenian suggestion is to hold that [NYC = NYC] is grounded in [It is essential to identity that everything is self-identical]—a fact that is not about derivative entities (or so one may suppose). Rosen suggested this proposal to me in personal communication.

10 For further suggestions about how identity facts might be grounded, see Shumener (2017, 2020).

11 In response to the account based on Sider’s conception of fundamentality, sceptics of all stripes about joint-carvingness will frown. Against the account based on Rosen’s view about the connection between essence and grounding, see Glazier (2017), where it is argued that it is not generally the case that [x is essentially such that p] grounds [p], and Spinelli (2021), where it is argued that nothing is essentially self-identical.

12 I focused on [NYC = NYC] only in order to keep the discussion short. Other kinds of facts could be invoked instead in the present context, for instance [NYC ∈ {NYC}], [Manhattan is part of NYC], and [To be a city is to be a city], where the form ‘To be φ is to be ψ’ is here understood as expressing so-called “generic” or “generalised” identity (about this non-standard form of identity, see Rayo 2013, Dorr 2016 and Correia & Skiles 2019).

13 Here and in the next sentence, I say ‘at least as challenging’ rather than ‘more challenging’, because I want to leave room for the view that all grounding facts are about derivative entities (see footnote 3).

14 A nice example is the following. Suppose, with Leuenberger (2020), that some facts are partially grounded without being fully grounded, and let F be such a fact. Assume that in Truism, ‘fundamental’ means not fully grounded, and that ‘less fundamental than’ is understood in such a way that ‘being partially grounded by implies being less fundamental than’ is true. Then F is a counterexample to Truism. Thanks to an anonymous reviewer for bringing this example to my attention, and to another anonymous reviewer for insisting that I discuss it in the paper.

15 The notion of absolute fundamentality that is naturally paired with the notion of relative fundamentality at work in the previous footnote is, I take it, that of not being partially grounded. If interpreted as involving these two notions, Truism is not falsified by the fact F invoked there.

16 [Note added after this paper has been accepted for publication.] I develop two further views in my “Fundamentality from Grounding Trees”, forthcoming in *Synthese*, which has been accepted for publication while the reviewing process for this paper was in its last stage. Both views are accounts of being more fundamental than in terms of grounding. One is extensionally equivalent to Werner’s. Principle LF → G (see below) is an immediate consequence of both accounts, and for that reason these accounts, just like Bennett’s account and Werner’s account, make the argument from Settledness dialectically ineffective.

17 I take it that those that can be formulated in terms of other building relations are irrelevant in the present context.

18 I also believe that the characterisation is problematic for various reasons, but I shall leave the issue aside.

19 The two characterisations actually also yield the converse of Truism: if G is non-fundamental, then by the first characterisation G is grounded, and hence some fact F at least partially grounds G; but then by clause (2) of the second characterisation, some fact is more fundamental than G. Thus, Bennett’s account delivers the nice-sounding principle that a fact is absolutely fundamental iff no fact is more fundamental than it.

20 One precisification that I specifically develop in the paper is the familiar modal precisification, on which entailment is understood as necessary implication and implication is understood as material implication. Although this precisification has the virtues of simplicity and familiarity, it is problematic in the context of Grounding from Fundamentality. I develop this point and suggest alternative precisifications in the paper.

21 Importantly, it was not initially assumed that *every* physical fact is fundamental. Making this assumption would go against the way I conceive of the interaction between relative fundamentality and the truth-functional operations. On my account, for instance, any true conjunction is less fundamental than both its conjuncts. Since conjunctions of physical facts are themselves physical facts, this principle and Truism together entail that some physical facts are non-fundamental.

22 A further, straightforward argument in favour of Settledness can be devised that invokes Bennett’s (2011) and deRosset’s (2013) view that grounding is “superinternal”, i.e. that every grounding fact [F is grounded in FF] is grounded in FF. Suppose that grounding is both factive and superinternal. Couple this assumption with the assumption that if a fact is grounded in other facts, then the former is less fundamental than the latter (the view is a consequence of Bennett’s, 2017 account of being more fundamental than (see above), and it has been stated or defended in many other places, e.g. Bennett, 2011, Koslicki, 2015, Rabin, 2018, Raven, 2012, Rosen, 2010 and Wilson, 2012). Then Settledness follows. Whatever its merits in other contexts, however, this argument—call it *the argument from superinternality*— is a non-starter in the context of assessing the argument from Settledness. The argument from superinternality is meant to support the conclusion of the argument from Settledness by supporting one of its premises. Now the former argument crucially involves the view that grounding is superinternal, and this view obviously entails the conclusion of the argument from Settledness. Therefore, using the argument from superinternality to support the argument from Settledness is question-begging.

23 Thanks to an anonymous reviewer for pressing me to discuss the argument along these lines. The counterexample presented below is theirs.

24 The presence of the qualification “immediate” is crucial. I clearly cannot endorse the principle without this qualification, as an example structurally similar to the counterexample to (B) discussed above shows. Suppose S and T are true, and that the fact expressed by T is much less fundamental than the fact expressed by S. The account I put forward predicts that the fact expressed by S is immediately more fundamental than the fact expressed by ˹S ∨ (T & ¬T)˺, and hence that the latter is *more* fundamental than the fact expressed by T.
